# Clinical Society of Maryland

**Published:** 1893-06

**Authors:** W. T. Watson

**Affiliations:** Sec’y; 1519 Broadway, Baltimore


### Society Reports.

### CLINICAL SOCIETY OF MARYLAND.

BALTIMORE, March 3, 1893.

The 277th regular meeting was called to order by the President, Dr. Wm. E. Moseley.

Dr. J. W. Chambers exhibited a patient upon whom he had operated in November, 1892, for injury caused by gunshot wound. The upper portion of the humerus and the lower end of the ulna were resected and two fingers amputated. About six inches of the upper end of the humerus was removed and three inches of the lower end of the ulna. It was a question of amputation or resection. Dr. Chambers thinks that most any condition of an arm is preferable to no arm. An arm is worth something for appearance sake even if it is not useful, and it helps to keep away the curve of the spine which occurs after the loss of an arm. From the statistics obtained during the civil war it would appear that there is somewhat greater danger from resection than from amputation. In six cases resected by Dr. Chambers there has been one death. This death occurred in a man of 70, who died of pneumonia not long after the operation. Recently five primary cases have been reported without a death. With the present methods resection will probably give as good or better results than amputation. This is so with the thigh and there is no reason why it should not be with the humerus. It is usually stated that when we remove more than four inches of the humerus we cannot expect a very useful arm. There is one case, however, reported in which six inches was removed and the man had a useful arm and could lift 200 pounds.

Regarding resection of the ulna, the text-books state where you have a gunshot wound or severe injury to the lower end of either radius or ulna it is better to amputate, or to resect an equal portion of both, otherwise you will have the hand left in a distorted position. The two cases shown by Dr. Chambers, show that this is not necessarily the case, because in neither of them is there any twisting or turning.

DR. JOSEPH H. BRANHAM said that these two cases illustrate very forcibly that under modern antiseptic methods, limbs can be saved which a short time ago would have been amputated without any hesitation. It would seem that, unless there is some injury besides that to the bone and shoulder, which makes amputation necessary, with antiseptic precautions, the results will be just as good in the partial excision of the upper end of the humerus as in amputation at the shoulder joint. Of course, if there is injury to the more important vessels then the excision would be improper and bad results might follow. Dr. Branham had treated a case somewhat similar to the one exhibited by Dr. Chambers. He saw him two weeks after the accident. The head of the humerus was destroyed and the fragments of bone that were loose were picked out with forceps. An abscess afterwards developed which opened up the axillary region very extensively. This was drained with a tube and irrigated. The boy made an excellent recovery and now has a good position as a clerk in a railroad office. Not much deformity has resulted.

DR. J. H. BRANHAM read a paper on

EXCISION OF THE PILE-BEARING MEMBRANE FOR HEMORRHOIDS—(WHITEHEAD'S OPERATION.)

An advance in the treatment of hemorrhoids was undoubtedly made in 1886, when Whitehead reported his method of total excision of the pile-bearing membrane to the British Medical Society. At that time he had operated on three hundred cases with uniformly good results. The cases which should be submitted to this operation are those severe ones in which the disease is extensive, involving the whole circumference of the mucous membrane lining the lower part of the rectum. Dr. Branham has employed the operation for over three years, and while the number of his cases is not large the results have been very satisfactory, and he strongly favors the method although it has been condemned by nearly all the prominent rectal specialists. The different steps in the operation were described. A 1-2000 solution of bichloride of mercury is the antiseptic used. The wound is closed by a continuous cat-gut suture carried through the skin and the tissues at the bottom of the wound and through the severed mucous membrane. Dr. Branham's cases number 15. In no instance has the hemorrhage been severe, and in none has there been secondary hemorrhage. No abscess or septic infection occurred in these cases. Union was primary in most of the cases, but a slight suppuration occurred in several instances. The advantages of this operation over other operations especially the ligature and the clamp and cautery, are, that the ligature leaves the wound open to granulate and is more liable to infection and secondary hemorrhage, is less thorough and is apt to be followed by a return. The clamp and cautery while leaving the wound at first aseptic is followed by a large granulation and consequently is more liable to cause contraction with symptoms of stricture.

DR. S. T. EARLE did not think that as a rule rectal specialists condemned the operation. They simply did not recommend it in all cases of hemorrhoids. They nearly all speak of it in high terms in cases for which it is especially adapted. Dr. Earle has done the operation repeatedly but only finds it necessary in about one out of six cases. He limits his cases to those where there is a varicose condition of the systemic veins together with internal hemorrhoids ; in this condition no other operation can take its place. He has had in one case, where there was considerable prolapse of the mucous membrane, together with the hemorrhoids, a return of the prolapse in one portion after excision by Whitehead's method. This prolapse was excised and there was no further trouble. Dr. Earle has never met with any hemorrhage to speak of in this operation and never had any secondary hemorrhage to occur. This operation is unnecessary in the large majority of cases. Where there are distinct and separate internal hemorrhoids it is much easier to excise them and the results are just as good.

DR. HUNTER ROBB took exception to the use of bichloride of mercury for disinfecting the rectal mucous membrane, and to the use of cat-gut as a suture material. From the uniformly fatal results produced by corrosive sublimate in irrigating the peritoneal cavity of dogs with solutions of 1-60000, he is inclined now to hesitate before employing this agent on mucous, serous or incised surfaces. It has been proven that corrosive sublimate in solution even as week as 1-60000 will produce a superficial necrosis of the tissues, and it has also been shown that even in strengths of 1-2000 or 1-3000 the drug is not always germicidal in its action. It would not seem advisable, therefore, to use a substance that causes destruction of the tissues, when in addition there is no certainty that the micro organisms present will be killed. By producing a necrosis of the tissues the normal resistance of the part would be interfered with and any virulent bacteria that might originally have been present or those that came subsequently in contact with the wounded surface would be much more likely to give rise to an infection of the part. He said that he had repeated with some modifications Dr. Halsted's experiments of irrigating the peritoneal cavity of dogs with bichloride of mercury. He had used 700 c. c. freshly made aqueous solutions of corrosive sublimate in strengths of 1-40000 and 1-60000. Immediately after using one of these solutions the peritoneal cavity was irrigated with the same quantity of sterile warm water, and then sponged as dry as possible. In from twelve hours to four or five days the animals all died. The lesions found at the autopsy were those produced by the toxic effects of corrosive sublimate. They consisted of marked diphtheritic deposits on the intestinal mucous membrane with intense hyperaemia, particularly in the large intestine and rectum. Although in some instances solutions of bichloride of mercury may be used in the peritoneal cavity without any unfavorable sequel, the results of those experiments with dilute solutions would necessarily lead one to be most careful, since the susceptibility of a given individual to its evil effects can never be predicted.

As to the use of cat-gut, it seemed to him that it had been clearly proven by the experiments made by Dr. Ghrisky and himself that this substance is a most favorable suture material for micro-organismal invasion, and besides it is impossible to be absolutely sure of sterilizing a cat-gut sufficiently strong to be of use. In their experience silkworm gut had proven to be the suture material most resistant to bacterial growth, and it has the advantage that it can be easily rendered sterile.

Whitehead's operation for hemorrhoids is one that he thought it rarely necessary to employ. The operation that he performs is that carried out by Dr. Kelly. The apex of the hemorrhoid is held with a pair of bullet forceps or by a tenaculum, and an incision made through the superficial layer of tissues encircling its base. A double ligature is passed through the centre of the hemorrhoid at its base, and each ligature tied separately in the line of incision, one anteriorly and one posteriorly. The portion of the tissues beyond the constriction is then cut off and the pedicle lightly cauterized. A simple sterile protective dressing is applied. This method of operation has given very satisfactory results.

DR. J. W. CHAMBERS thought that Dr. Branham's cases showed at least that good results can be obtained by Whitehead's operation. He usually does Smith's operation with clamp and cautery. The matter of putting on a ligature and then using a cautery, as described by Dr. Robb, is doing two operations instead of one. The clamp and cautery is unquestionably the best method in simple cases. As to washing out the rectum with bichloride solution, that is hardly as dangerous as Dr. Robb has suggested. It is hard to draw comparisons between the effects of flushing out the peritoneal cavity of a dog and the irrigation of the rectum that has been subject to severe congestion. Of course, it is impossible to make the rectum or the mouth perfectly aseptic, but there is something about these two localities that makes their tissues unusually resistant to infection and wounds in these regions usually do particularly well.

DR. W. S. GARDNER thought that there was something of a scare about bichloride poisoning. Leopold keeps his sponges in 1-10000 bichloride and swabs out the abdominal cavity with the solution. During a Caesarian section he has seen Leopold swab out the uterus and wipe off the outside of that organ with this solution, and the patient did perfectly well. Dr. Gardner has a great many times washed out the dilated puerperal uterus with a solution of 1–4,000 bichloride with no trouble resulting. Certainly the dilated uterus and vagina offer a very much larger surface for absorption than you can get in the most distended rectum.

DR. WM. E. MOSELEY thought that it was hardly proper to make comparisons between bichloride that is absorbed through the peritoneal membrane into the circulation and then produces lesions in the rectum, and the effects due to irrigating the surface of the rectum. He does not use bichloride in the peritoneal cavity but has seen it used repeatedly with no evil results. The method of transfixion as described by Dr. Robb, was the oldest one that Dr. Moseley had ever seen or practiced. It is a very good method but not new.

DR. EARLE said that he did not hesitate to use bichloride in the rectum, but always takes the precaution to wash it out afterwards with sterilized water. He had once seen a diarrhoea after the use of bichloride. We cannot expect to make the rectum aseptic but should try to make it as nearly so as possible.

DR. BRANHAM named Matthews, Kelsey and Allingham, as amongst those who condemned the Whitehead operation. As to the use of bichloride, Dr. Robb says that $\frac{1}{8}$ of a grain will kill a dog. Human beings with syphilis take $\frac{1}{8}$ of a grain three or four times a day without any evil results. The peritoneum is an absorbing membrane, and if much bichloride solution is left in it death may result, but the lower portion of the rectum is lined with pavement epithelium and histologically does not differ from skin and there is probably no more likelihood of poisoning from bichloride in applying it to this tissue than in applying it to the skin. This portion of the rectum is an excreting and not an absorbing tissue. As to the use of cat-gut, there seems to be some extra dangers of infection from it. The disadvantages of silk is that it has to be removed and causes some irritation.

DR. HUNTER ROBB read a paper on "Anæsthesia in Gynæcological Examinations."

1519 Broadway, Baltimore. W. T. WATSON, Sec'y.

